# Indomethacin-induced activation of the death receptor-mediated apoptosis pathway circumvents acquired doxorubicin resistance in SCLC cells

**DOI:** 10.1038/sj.bjc.6602516

**Published:** 2005-04-05

**Authors:** D J A de Groot, T Timmer, D C J Spierings, T K P Le, S de Jong, E G E de Vries

**Affiliations:** 1 Department of Medical Oncology, University Medical Center Groningen, PO Box 30.001, 9700 RB, Groningen, The Netherlands

**Keywords:** GLC_4_, Bid, mitochondria

## Abstract

Small-cell lung cancers (SCLCs) initially respond to chemotherapy but are often resistant at recurrence. A potentially new method to overcome resistance is to combine classical chemotherapeutic drugs with apoptosis induction via tumour necrosis factor (TNF) death receptor family members such as Fas. The doxorubicin-resistant human SCLC cell line GLC_4_-Adr and its parental doxorubicin-sensitive line GLC_4_ were used to analyse the potential of the Fas-mediated apoptotic pathway and the mitochondrial apoptotic pathway to modulate doxorubicin resistance in SCLC. Western blotting showed that all proteins necessary for death-inducing signalling complex formation and several inhibitors of apoptosis were expressed in both lines. The proapototic proteins Bid and caspase-8, however, were higher expressed in GLC_4_-Adr. In addition, GLC_4_-Adr expressed more Fas (3.1x) at the cell membrane. Both lines were resistant to anti-Fas antibody, but plus the protein synthesis inhibitor cycloheximide anti-Fas antibody induced 40% apoptosis in GLC_4_-Adr. Indomethacin, which targets the mitochondrial apoptotic pathway, induced apoptosis in GLC_4_-Adr but not in GLC_4_ cells. Surprisingly, in GLC_4_-Adr indomethacin induced caspase-8 and caspase-9 activation as well as Bid cleavage, while both caspase-8 and caspase-9 specific inhibitors blocked indomethacin-induced apoptosis. In GLC_4_-Adr, doxorubicin plus indomethacin resulted in elevated caspase activity and a 2.7-fold enhanced sensitivity to doxorubicin. In contrast, no effect of indomethacin on doxorubicin sensitivity was observed in GLC_4_. Our findings show that indomethacin increases the cytotoxic activity of doxorubicin in a doxorubicin-resistant SCLC cell line partly via the death receptor apoptosis pathway, independent of Fas.

Lung cancer is the tumour type with the highest incidence in the Western world. A total of 25% of lung cancers are of the small-cell lung cancer (SCLC) type. These tumours are well known for their initial sensitivity to chemotherapeutic agents and thereafter frequently recur at which time the tumour is drug resistant (Glisson, 2003). A common mechanism for drug resistance shared by chemotherapeutic drugs is the failure of the tumour cells to go into apoptosis. Interestingly, tumour cells have an independent pathway available, which can be used to induce apoptosis, namely, the death receptor ligand signalling pathway (Younes and Kadin, 2003). This has raised interest to exploit this pathway to circumvent drug resistance. Fas and Fas ligand (FasL) belong to the TNF family of death receptors and ligands (Trauth *et al*, 1989; Itoh *et al*, 1991; Suda *et al*, 1993). Fas expression is present in many tumours and tumour cell lines including in SCLC (Friesen *et al*, 1997; Muller *et al*, 1997; Niehans *et al*, 1997; Fulda *et al*, 1998; Kawasaki *et al*, 2000). After trimerisation of Fas on the cell membrane by extracellular FasL (Huang *et al*, 1996), Fas-associated Death Domain (FADD) and caspase-8 bind to the intracellular death domains of Fas and induce a death signal in the cell (Debatin *et al*, 1997). This leads to the activation of a cascade of caspases and eventually to cell death. In addition, several antiapoptosis proteins regulate the Fas-mediated death pathway. Important antiapoptosis proteins are decoy receptor 3 (DcR3), Fas-associated phosphatase-1 (FAP-1), the long and short isoform of FLICE-inhibitory protein (FLIP_1_ and FLIP_S_) and the inhibitors of apoptosis family (IAPs) (Sato *et al*, 1995; Deveraux *et al*, 1997; Yanagisawa *et al*, 1997; Pitti *et al*, 1998; Li *et al*, 2000).

There is an alternative pathway for death receptor-induced apoptosis that involves the mitochondria (Scaffidi *et al* 1998, 1999). This pathway is controlled by proapoptotic and antiapoptotic proteins from the Bcl-2 family. One of the key proapoptotic proteins in this pathway is Bid. When caspase-8 is activated in the initial phase of death receptor-induced apoptosis, it can cleave Bid. The p15 form of truncated Bid (tBid) translocates to the mitochondria where cytochrome *c* is released. Cytochrome *c* activates caspase-9, which activates downstream effector caspases resulting in apoptosis (Luo *et al*, 1998).

In several tumour cell lines, including SCLC cell lines, Fas membrane expression is upregulated after exposure to chemotherapeutic agents (Friesen *et al*, 1999). This can enhance sensitivity to apoptosis-inducing anti-Fas antibody. Therefore, induction of Fas-mediated apoptosis together with chemotherapy may be an option to overcome drug resistance. At the moment, the major problem of FasL or stimulating anti-Fas antibody is the liver toxicity observed in mice (Ogasawara *et al*, 1993). However, several attempts are ongoing to circumvent liver toxicity.

Another option to modulate drug resistance is the inhibition of expression of antiapoptotic members of the Bcl-2 family of apoptosis with nonsteroidal anti-inflammatory drugs (NSAIDs). These drugs act by cyclooxygenase (COX) inhibition but can also affect death receptor-mediated apoptosis pathways (Bagrij *et al*, 2001) and induce apoptosis by downregulation of Bcl-2 family members (Liu *et al*, 1998, Crosby and DuBois, 2003). In SCLC cell lines, Bcl-2 family members have been described as important factors in chemotherapeutic drug resistance and therefore downregulation of Bcl-2 family members with an NSAID can be an interesting modality to circumvent drug resistance (Sartorius and Krammer, 2002). Human lung adenocarcinoma cells, exposed to NSAIDs showed an effective reduction of the antiapoptosis Bcl-2 family member Mcl-1 (Lin *et al*, 2001).

In this study, we investigated the possibility of utilising the Fas-mediated apoptosis route and indomethacin to modulate doxorubicin resistance in an acquired doxorubicin resistant SCLC cell line.

## MATERIALS AND METHODS

### Cell lines

GLC_4_ was derived from a pleural effusion in our laboratory and kept in culture in RPMI 1640 medium supplemented with 10% heat inactivated fetal calf serum (FCS) (both from Life Technologies, Breda, The Netherlands). GLC_4_-Adr obtained resistance to doxorubicin, but also to a wide range of other chemotherapeutic agents, by stepwise increasing concentrations of doxorubicin (Zijlstra *et al*, 1987; de Jong *et al*, 1990; Meijer *et al*, 1991; Versantvoort *et al*, 1995). GLC_4_-Adr is 190.6±16.2 times more resistant to doxorubicin than its parental cell line. The doxorubicin resistance in GLC_4_-Adr is due to a downregulation of the activity of DNA-topoisomerase II (TOPO II) and amplification and overexpression of the *MRP-1* gene GLC_4_-Adr was exposed to 1.2 *μ*M doxorubicin twice weekly. GLC_4_-Adr was cultured without doxorubicin for 20 days prior to experiments. Cells were incubated at 37°C in a humidified atmosphere with 5% CO_2_. Cells from exponentially growing cultures were used for all experiments.

### Antibodies and reagents

The antibodies used for Western blot analysis were all diluted in Tris buffered saline (TBS) buffer (20 mM Tris-HCl, 137 mM NaCl_2_ and 0.05% Tween 20) supplemented with 5% skim milk powder (Merck, Darmstadt, Germany). The anti-FasL-, FADD- and XIAP antibody were purchased from Transduction Laboratories (Alphen a/d Rijn, the Netherlands), the Fas-, Bax-, Bcl-2-, Bcl-X_S/L_- and FAP-1 antibody from Santa Cruz (Heerhugowaard, the Netherlands), the caspase-8 antibody from Cell Signalling (Leusden, the Netherlands). The caspase-9, caspase-3- and FLIP antibody were obtained from Pharmingen (Alphen a/d Rijn, the Netherlands). The Mcl-1 antibody was purchased from DAKO (Glostrup, Denmark). The Bid antibody was kindly provided by Dr J Borst, the Netherlands Cancer Institute, Amsterdam. The PARP antibody was obtained from Roche (Almere, the Netherlands) and the COX-2 antibody was obtained from Cayman Chemical (Veenendaal, the Netherlands). The anti-mouse secondary antibody was a horseradish peroxidase-labelled rabbit anti-mouse, which was diluted 1 : 1500 in TBS supplemented with 5% milk. The secondary anti-rabbit antibody, a swine anti-rabbit, was diluted 1 : 1500. Against goat primary antibodies, a rabbit anti-goat horseradish peroxidase-labelled antibody 1 : 2000 was used. The fluorescein (FITC) coupled rabbit anti-mouse antibody was diluted 1 : 20. All secondary antibodies were purchased from DAKO (Glostrup, Denmark). The proapoptotic mouse anti-Fas antibody 7C11 was obtained from Immunotech (Versailles, France) and the phycoerythrin (PE)-labelled anti-human Fas DX2 and the anti-FasL antibody NOK-1 antibody from Pharmingen, Alphen a/d Rijn, the Netherlands. The mouse monoclonal CH11 anti-Fas antibody (Upstate Biotechnology, Veenendaal, The Netherlands) was used for confocal laser microscopy. Doxorubicin was obtained from Pharmacia Upjohn (Woerden, The Netherlands). Indomethacin was purchased from ICN Biomedicals (Aurora, OH, USA), 3-[4,5-dimethylthiazol-2-yl]-2,5-diphenyltetrazolium bromide (MTT), cycloheximide from Sigma Aldrich (Zwijndrecht, The Netherlands), Ponceau S from Sigma-Aldrich and Coomassie blue solution was purchased from Biorad (Veenendaal, The Netherlands). MK-571 was purchased from Merck Sharp (Kirkland, Canada)

### SDS gel electrophoresis and Western blot

Proteins for Western blot analysis were extracted by lysing cells with sample buffer containing 0.125 M Tris-HCl, 2% SDS, 10% glycerol and 0.001% bromophenol blue. Samples were boiled for 5 min. An amount of 10 *μ*g protein was run on 10% SDS polyacrylamide gels at 200 V and transblotted onto polyvinylidene difluoride membranes (PVDF) (Millipore, Bedford, UK) with a semi-dry blot system. Equal protein loading was confirmed by Ponceau red staining of membranes and Coomassie blue staining of the gels. Membranes were activated in methanol for 5 min and washed three times with H_2_O and once with TBS without Tween 20. Membranes were then blocked for 1 h in TBS supplemented with 5% skim milk and probed with the primary antibody for 1 h. Membranes were washed three times with TBS and incubated with the horseradish peroxidase-bound secondary antibody for 1 h at room temperature. Membranes were washed three times with TBS and bands were visualised with chemoluminescence POD or Lumi-light^+^ (Roche Diagnostics, Basel, Switzerland). All experiments were performed three times.

### Confocal laser microscopy

The intracellular localisation of Fas in the cell lines was determined with confocal laser microscopy. Cells were washed cells once with RPM3 1640 medium 10% FCS. Glass slides were coated with 0.1% poly-L-lysine and dried at room temperature. A volume of 50 *μ*l of 4 × 10^5^ cells/ml were put on glass slides and left to adhere to the slides for 1 h. Cells were fixed with 4% paraformaldehyde in phosphate-buffered saline (PBS: 6.4 mM Na_2_HPO_4_, 15 mM KH_2_PO_4_; 0.14 mM NaCl; 2.7 mM KC1; pH 7.2) supplemented with 3.3 mM CaCl_2_ for 15 min. Cells were washed twice with PBS and incubated for 1 h with the CHl 1 anti-Fas antibody. After incubation with the primary antibody, cells were washed twice and incubated with a FITC coupled rabbit anti-mouse antibody for 30 min and washed twice before they were analysed on a Leica confocal laser microscope.

### Flow cytometry

To determine Fas membrane expression cells were harvested from the culture medium by centrifugation at 110 **g** for 5 min and washed twice with cold PBS supplemented with 2% FCS and 0.1% sodium azide. Cells were then incubated for 1 h with the PE-labelled anti-human Fas DX2 antibody, which was diluted 1 : 10 in cold PBS supplemented with 2% FCS and 0.1% sodium azide for 1 h on ice in the dark and washed twice with cold PBS. Analysis was performed on a Coulter Elite Flow cytometer (Becton Dickinson, Mount View, CA, USA) with Winlist and Winlist 32 software (Verity Software House, Inc., Topsham, ME, USA). Fas membrane expression was determined as mean fluorescence intensity (MFI). To study also the effect of indomethacin on Fas membrane expression, cells were incubated for 24 h with indomethacin. These experiments were performed three times.

### Isolation of total cellular RNA, cDNA synthesis and RT–PCR

RNA was isolated from log phase cultures of the cell lines. Cells were harvested by centrifugation at 110 **g** for 5 min and washed with PBS. RNA was isolated with the guanidine isothiocyanate method. An amount of 5 *μ*g RNA was treated with 20–100 U DNAse I (Roche Diagnostics Basel, Switzerland) for 30 min. Single-stranded cDNA was synthesised using M-MLV Reverse Transcriptase (Invitrogen Merelbeke, Belgium) and oligo dT primers according to the manufacturer's protocol. For RT–PCR 1 *μ*l of cDNA was used as the target in a total volume of 50 *μ*l. Reactions were performed according to standard protocols using the following primers and conditions. FasL (290 bp, 53°C), upstream 5′-CCTCCAGGCAGGCACAGTTCTTCC-3′ and downstream 5′-ATCTGGCTGGTAGACTCTCG-3′; Fas (338 bp, 49°C), upstream 5′-CATGGCTTAGAAGTGGAAAT-3′ and downstream 5′-ATTTATTGCCACTGTTTCAGG-3′, FADD (250 bp, 54°C), upstream 5′-AGCTCAAAGTCTCAGCACACC-3′ and downstream 5′-TCTGAGTTCCATGACATCGG-3′; Caspase-8 (355 bp, 54°C), upstream 5′-CTGCTTCATCTCTGTATCC-3′ and downstream 5′-GCAAAGTGACTGGATGTACC-3′; DcR3 (263 bp, 62°C), upstream 5′-AGCACGCATCGTGTCCACC-3′ and downstream 5′-GACGGCACGCTCACACTCC-3′; FAP-1 (276 bp, 54°C), upstream 5′-GGAGTTAGTCTAGAAGGAGC-3′ and downstream 5′-ACTGAATCCTAGACCTGAGC-3′; FLIP_long_ (262 bp, 54°C), upstream 5′-GAACATCCACAGAATAGACC-3′ and downstream 5′-GTATCTCTCTTCAGGTATGC-3′; FLIP_short_ (172 bp, 54°C), upstream 5′-GAACATCCACAGAATAGACC-3′ and downstream 5′-TTTCAGATCAGGACAATGGG-3′. All experiments were performed three times.

### Mutation screening of Fas

DNA was extracted from the cell lines using a standard laboratory technique. The *Fas* gene was screened for mutations by denaturing gradient gel electrophoresis of the extracted DNA. The entire coding region, including all splice site junctions, was amplified in 10 amplicons using primers and conditions as described earlier (Gronbaek *et al*, 1998). The amplicons were electrophoresed in a 9% polyacrylamide denaturing gradient gel containing 5% glycerol and 20–60% urea-formamide (100% urea-formamide=7 M urea and 40% deionised formamide). The gels were stained with ethidium bromide and photographed under an UV transilluminator.

### Apoptosis assay

Cells (1.5 × l0^4^ per well) were cultured in 96-well plates and optionally preincubated with 1 *μ*g ml^−1^ cycloheximide for 2 h. Apoptosis was induced by adding the anti-Fas antibody 7C11 (1 *μ*g ml^−1^) for 24 h. To determine whether indomethacin induces apoptosis, cells were incubated with different concentrations of indomethacin. To investigate whether apoptosis induction with indomethacin is Fas-mediated, cells were optionally preincubated with 2 *μ*g ml^−1^ NOK-1 and incubated with indomethacin for 24 h thereafter. Apoptosis was defined as the appearance of apoptotic bodies and/or chromatin condensation, using a fluorescence microscope. Results were expressed as the percentage of apoptotic cells in a culture by counting at least 200 cells per well. All apoptosis assays were performed three times in two-fold.

### Inhibition of indomethacin-induced apoptosis

At 1 h prior to indomethacin exposure, cells were incubated with 20 *μ*M broad-spectrum caspase inhibitor zVAD-fmk, caspase-8 inhibitor zIETD-fmk or caspase-9 inhibitor zLEHD-fmk (all from Calbiochem, Breda, The Netherlands). Cells were exposed to the combination of indomethacin and caspase inhibitor for 24 h after which acridine orange was added and the percentage apoptotic cells was calculated. Results are expressed as the percentage of apoptotic cells in a culture by counting at least 200 cells per well. All apoptosis assays were performed three times in two-fold.

### Caspase-3 activation assay

The cleavage assay was carried out in six-well plates according to Thornberry *et al* (2000). Activity of caspase-3 was assayed according to the manufacturer's instructions using the fluorescence peptide substrate Ac-DEVD-AFC (Biomol Tebu-bio, Heerhugowaard, The Netherlands). Fluorescence from free 7-amino-4-trifluoromethyl coumarin (AFC) was monitored in a FL600 Fluorimeter Bio-tek plate reader (Beun de Ronde, Abcoude, The Netherlands) using 380 nm excitation and 508 nm emission wavelengths. Relative caspase-3 activity was calculated by the fluorescence of a sample of treated cells by a sample of untreated cells. Protein from all samples was isolated to confirm apoptosis with PARP cleavage on Western blot. Experiments were performed three times.

### 3-[4,5-dimethylthiazol-2-yl]-2,5-diphenyltetrazolium bromide (MTT) assay

The cell lines were cultured in HAM/F12 and DMEM medium (1 : 1) (Life Technologies) supplemented with 20% FCS. The effect of doxorubicin and indomethacin on survival was tested MTT assay as described previously (Timmer-Bosscha *et al*, 1989). Cells were incubated for 4 days at 37°C and 5% CO_2_ in a humidified environment with a range of indomethacin concentrations and 10 and 2000 nM doxorubicin for GLC_4_ and GLC_4_-Adr respectively. The modulating effect of indomethacin (10 and 20 *μ*M) and MK-571 (50 *μ*M) on cell survival by doxorubicin were also tested in the MTT assay using continuous incubation. After a 4-day culture period, MTT (5 mg ml^−1^ in PBS) was added and formazan crystal production was measured as described previously. Controls consisted of media without cells (background extinction) and cells incubated with medium instead of chemotherapeutic agents. Experiments were performed three times in quadruplicate.

### Statistics

All experiments were performed at least three times on different occasions. Analysis included double-sided nonpaired *t*-test. A *P*-value <0.05 was considered significant.

## RESULTS

### Differences between the Fas-mediated apoptosis pathway of GLC_4_ and GLC_4_-Adr

**Figure 1:**
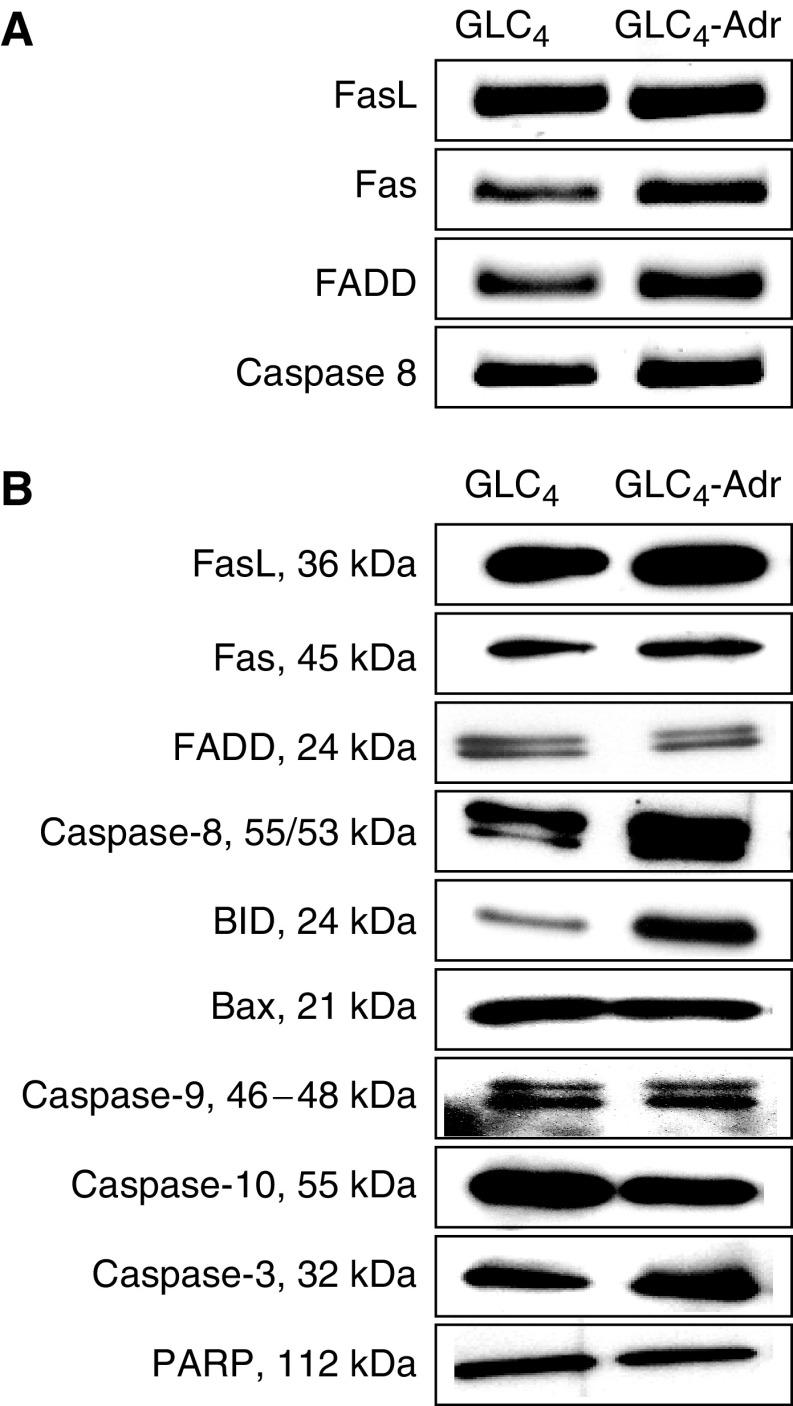
Basic mRNA and protein expression levels of proapoptotic proteins in GLC_4_ and GLC_4_-Adr in the Fas-mediated apoptosis pathway were determined at the mRNA level (**A**) and protein level (**B**). Representative examples of three independent experiments are shown.

The genes encoding the proapoptotic proteins FasL, Fas, FADD, and caspase-8 were all expressed at the mRNA level in GLC_4_ and GLC_4_-Adr (Figure 1A). The proteins FasL, Fas, FADD, caspase-8, Bid, caspase-9, caspase-3 and PARP were also present in both cell lines (Figure 1B). GLC_4_ contained less Bid and caspase-8 compared to GLC_4_-Adr, while there were no differences in the protein expression of FasL, Fas, FADD, Bax, caspase-9, caspase-10, caspase-3, and PARP between the two lines (Figure 1B). Mutation analysis of the entire *Fas* gene revealed no aberrant patterns in both cell lines.

**Figure 2:**
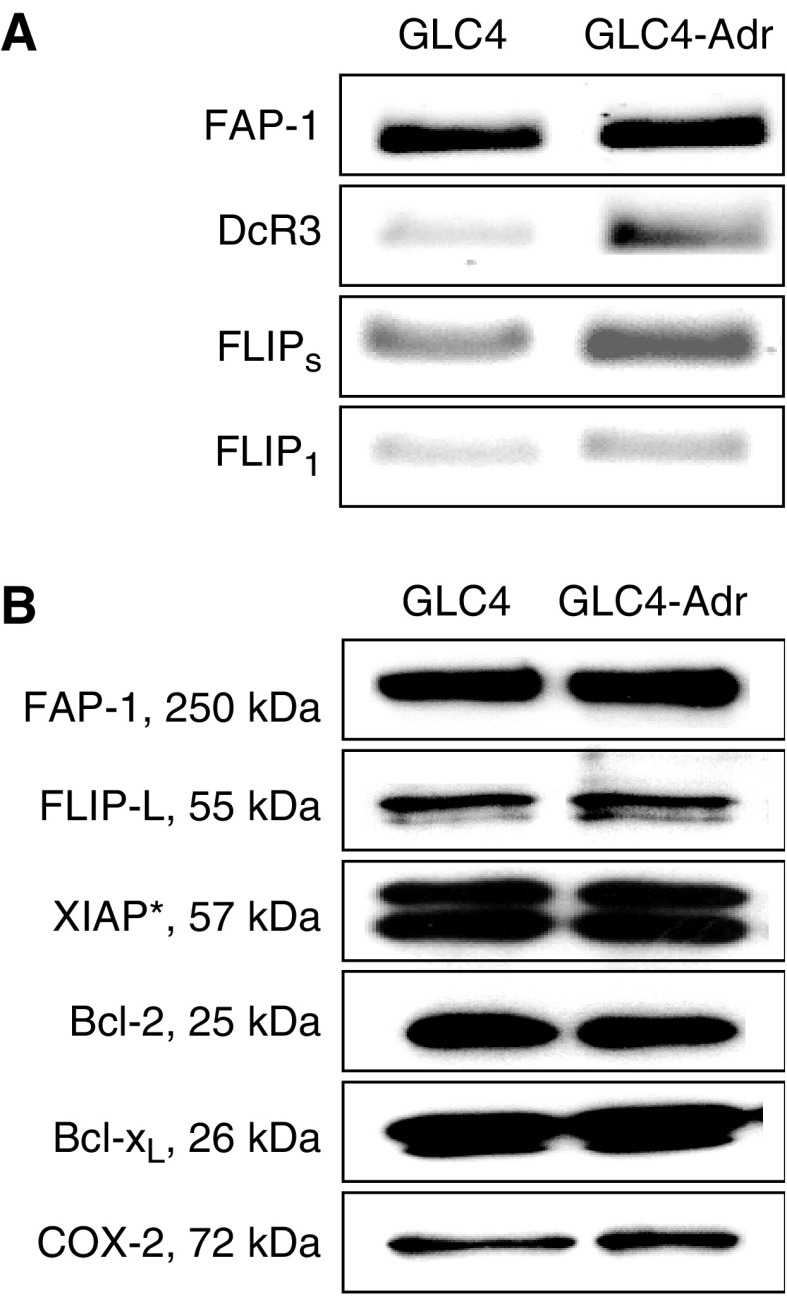
Basic mRNA and protein expression levels of antiapoptotic proteins in GLC_4_ and GLC_4_-Adr in the Fas-mediated apoptosis pathway were determined at the mRNA level (**A**) and protein level (**B**). Representative examples of three independent experiments are shown.

Antiapoptosis genes were present in GLC_4_ and GLC_4_-Adr. RT–PCR analysis revealed a higher expression of FLIP_1_, FLIP_S_ and DcR3 in GLC_4_-Adr compared to GLC_4_ (Figure 2A). Western blot analysis showed no differences in expression of the apoptosis inhibitors FAP-1, FLIP, Bcl-2, Bcl-X_L_ and XIAP between the cell lines. The nonspecific anti-XIAP and Bc1-X_L_, immunoreactive molecule as indicated (^*^) served as an internal loading control (Deveraux *et al*, 1999, Delhalle *et al*, 2002) (Figure 2B). There were also no differences in COX-2 expression (Figure 2B).

**Figure 3:**
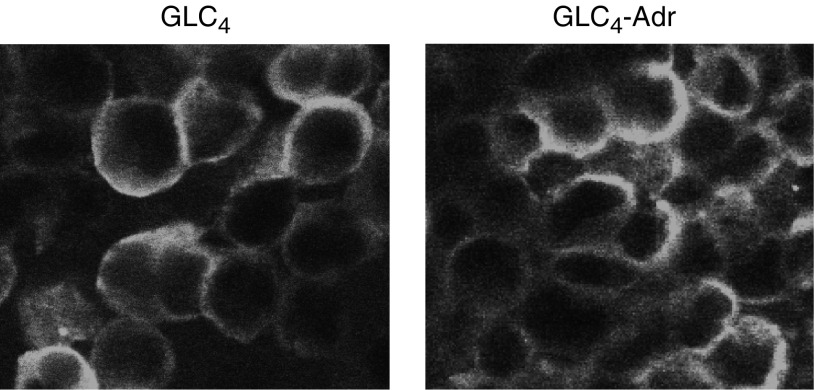
Fas localisation in GLC_4_ and GLC_4_-Adr determined with the mouse monoclonal CH11 anti-Fas antibody (Upstate Biotechnology) using confocal laser microscopy.

Confocal laser microscopy revealed that in both cell lines Fas was present in the cytoplasm and at the cell membrane (Figure 3). However, as determined by flow cytometry GLC_4_-Adr contained 3.1-fold more Fas on the cell membrane than GLC_4_. MFI were on average 15.4 in GLC_4_ and 47.5 in GLC_4_-Adr.

### Anti-Fas antibody induces apoptosis

**Figure 4:**
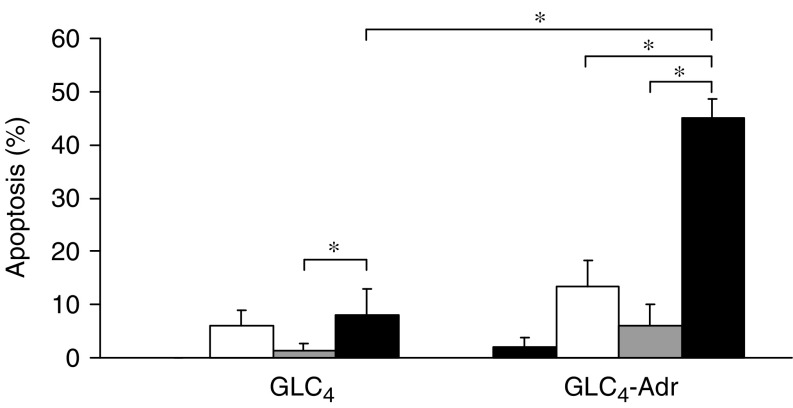
Fas-mediated apoptosis induction. Apoptosis induction in GLC_4_ and GLC_4_-Adr was determined after exposure to medium (stripes), cycloheximide (white), anti-Fas antibody 1 *μ*g ml^−1^ (grey) or both (black) using the apoptosis assay. Data represent the mean±s.d. of three independent experiments (^*^P<0.05).

**Figure 5:**
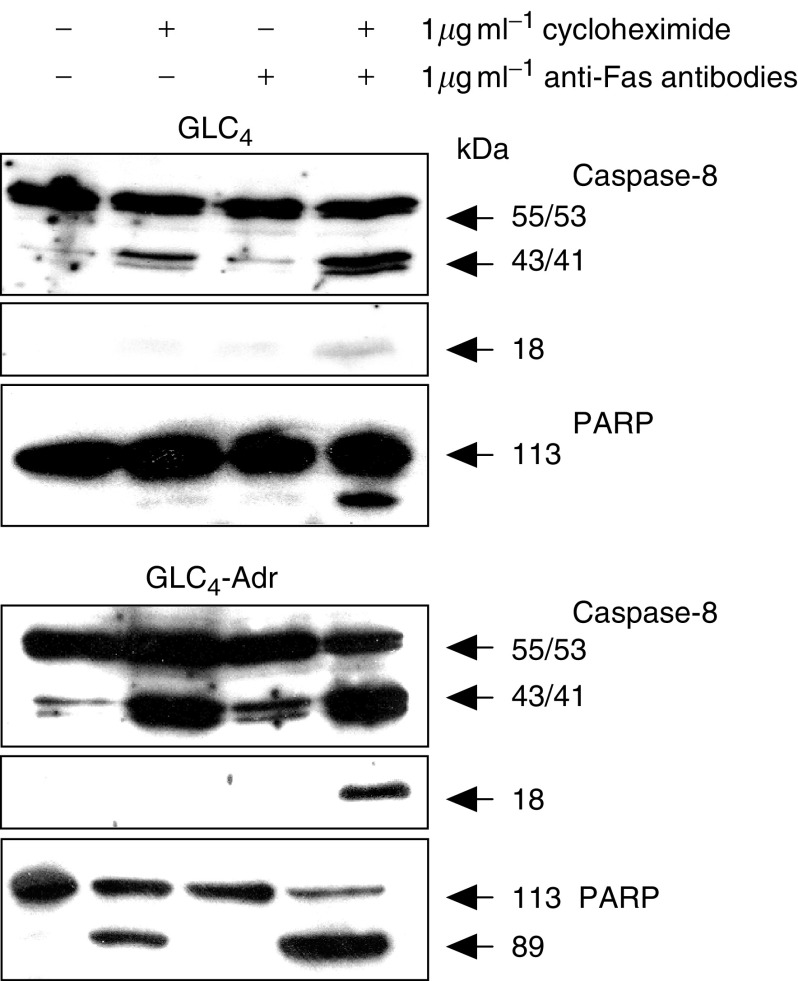
Fas-mediated caspase cleavage. PARP and caspase-8 cleavage were determined after exposing GLC_4_ and GLC_4_-Adr to anti-Fas antibody for 24 h and cycloheximide for 24 and 2 h preincubation. Representative example of three independent experiments.

Functionality of the Fas pathway was tested by exposure to anti-Fas antibody (24 h) and a combination of anti-Fas antibody (24 h) and cycloheximide (2 h preincubation). The anti-Fas antibody alone hardly induced apoptosis. Cotreatment with cycloheximide largely increased apoptosis in GLC_4_-Adr (40%) but had almost no effect in GLC_4_ (8%) (Figure 4). Caspase-8 activation was used as intracellular determinants for activation of the Fas pathway and PARP cleavage as a marker for apoptosis. Surprisingly, no effect of anti-Fas antibody alone on caspase-8 cleavage was found. Intermediate cleavage products of caspase-8 (p41/p43) were detected after exposure to cycloheximide alone or in combination with anti-Fas antibody in both cell lines (Figure 5). Active caspase-8 (pi8 product) was especially observed in GLC_4_-Adr but only after cotreatment with cycloheximide and anti-Fas antibody. These results indicate the presence of intracellular inhibitors) of the Fas-mediated apoptosis pathway, presumably at the level of caspase-8.

### Indomethacin induces apoptosis in GLC_4_-Adr but not in GLC_4_

**Figure 6:**
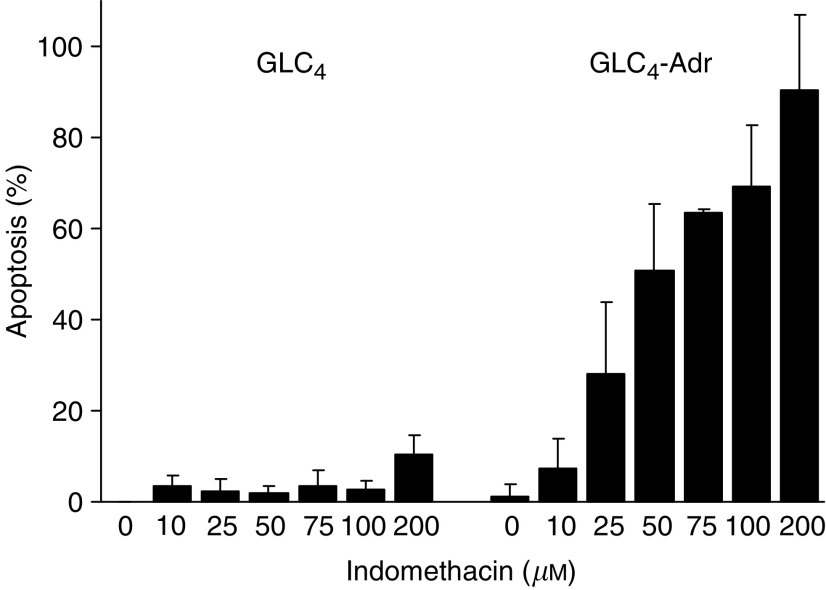
Indomethacin-mediated apoptosis. Apoptosis induction in GLC_4_ and GLC_4_-Adr was determined after exposure to different concentrations of indomethacin for 48 h.

**Figure 7:**
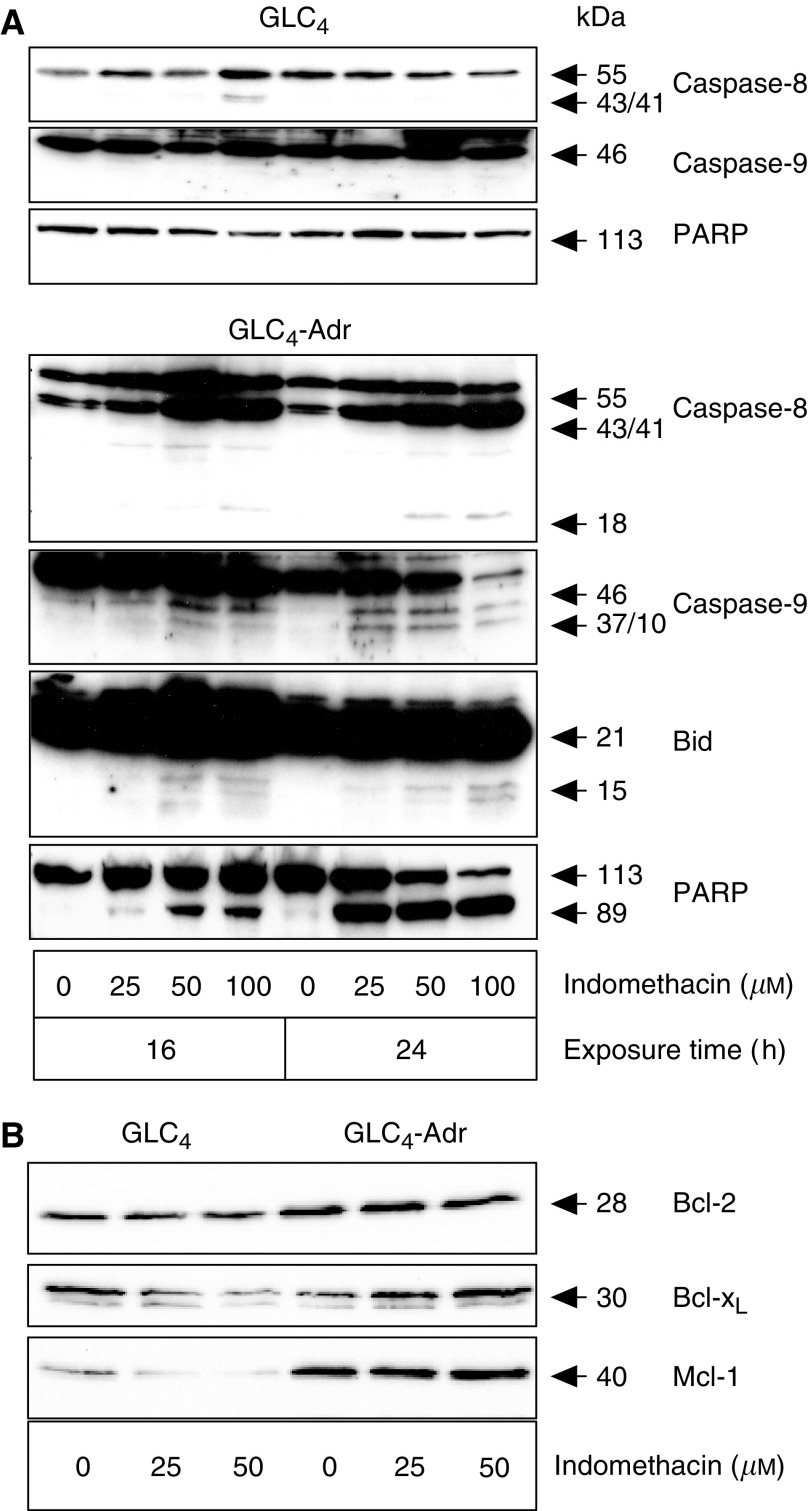
(**A**) Indomethacin-induced activation of the death receptor apoptosis pathway. Caspase-8, Bid and PARP cleavage were determined after exposing GLC_4_ and GLC_4_-Adr to 0, 25, 50 and 100 *μ*M indomethacin for 16 and 24 h. Representative example of three experiments are shown. (**B**) Expression of Bcl-2, Bc1-X_S/L_ and Mcl-1 after 24 h of 25 and 50 *μ*M indomethacin exposure.

Indomethacin alone had hardly any effect on apoptosis induction in GLC_4_ but already induced apoptosis (28%) in GLC_4_-Adr at 25 *μ*M (Figure 6). GLC_4_ and GLC_4_-Adr were exposed to 0, 25, 50 and 100 *μ*M indomethacin for 16 and 24 h in order to study the apoptosis-inducing effect of indomethacin in more detail. Cleavage of caspase-8, Bid and PARP was investigated with Western blotting. Caspase-8, Bid, caspase-9 and PARP activation in GLC_4_-Adr occurred 16 h after the addition of 50 *μ*M indomethacin. In GLC_4_-Adr 100 *μ*M indomethacin induced clearly detectable levels of activated caspase-8 (p 18) as well as massive cleavage of full-length caspase-8 and PARP. Indomethacin more effectively induced caspase-8 activation than the combination of anti-Fas antibody and cycloheximide in GLC_4_-Adr. However, even at these high indomethacin concentrations, no activation of caspase-8, Bid and PARP was observed in GLC_4_ (Figure 7A).

To further investigate the mechanism by which indomethacin induces apoptosis, GLC_4_ and GLC_4_-Adr cells were exposed to 25 and 50 *μ*M indomethacin and protein expression levels of anti-apoptotic Bcl-2 family members were analysed. No changes in Bcl-2, Bcl-X_S/L_ or Mcl-1 expression were observed in both cell lines (Figure 7B).

**Figure 8:**
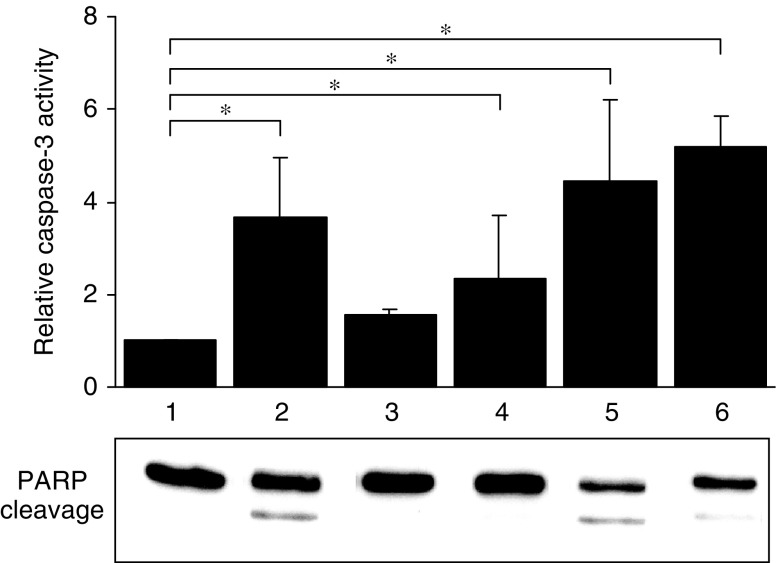
Effects of modulators on caspase-3 activation in GLC_4_-Adr after 48 h of exposure to. (1) medium, (2) indomethacin (25 *μ*M), (3) doxorubicin (3 *μ*M), (4) anti-Fas antibody (1 *μ*g ml^−1^), (5) anti-Fas antibody and doxorubicin (3 *μ*M), (6) indomethacin (25 *μ*M) and doxorubicin (3 *μ*M). Exposure to the anti-Fas antibody was only for the last 24 h. Data represent the mean±s.d. of three independent experiments (^*^P<0.05).

To determine more quantitatively the effect of various drug combinations on apoptosis in GLC_4_-Adr, caspase-3 activation was measured with the DEVD-AFC cleavage assay. Doxorubicin alone showed minimal caspase-3 activation. Doxorubicin in combination with anti-Fas antibody had a slightly additive effect on caspase-3 activation. The combination of doxorubicin with indomethacin was, however, the most effective combination to induce caspase-3 activation (Figure 8).

### Indomethacin induces caspase-8 and caspase-9 activation independently from Fas

The nature of the indomethacin-induced caspase-8 activation was further investigated. Apoptosis induction in GLC_4_-Adr could not be prevented by preincubation with the anti-FasL NOK-1 antibody (data not shown). This means that indomethacin-induced apoptosis is not caused by autocrine or paracrine Fas/FasL interactions. In addition, indomethacin at a concentration of 50 *μ*M for 24 h did not affect Fas membrane expression (results not shown).

**Figure 9:**
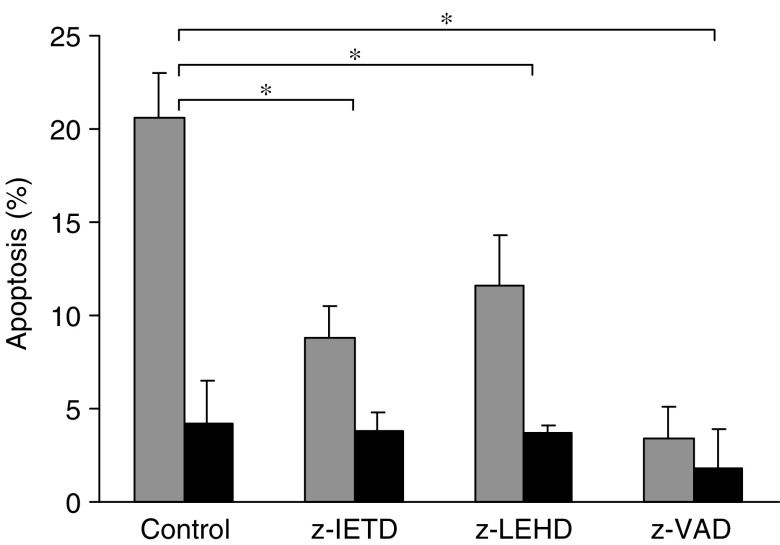
Inhibition of indomethacin-induced apoptosis. Apoptosis induction in GLG_4_-Adr after exposure to 50 *μ*M indomethacin (grey) or 0 *μ*M indomethacin (black) in combination with the caspase-8 inhbitor zIETD-fmk, the caspase-9 inhibitor zLEHD or the broad-spectrum caspase inhibitor zVAD-fmk for 24 h.

Exposure of GLC_4_-Adr cells to indomethacin in combination with caspase inhibitors revealed that indomethacin-induced apoptosis is reduced when cells are exposed to indomethacin in combination with zIETD-fmk, zLEHD-fmk or zVAD-fmk activity. The caspase-8-specific inhibitor zIETD-fmk and the caspase-9-specific inhibitor zLEHD-fmk reduced indomethacin-induced apoptosis by 58 and 44%, respectively, the broad-spectrum caspase inhibitor zVAD-fmk by 84% (Figure 9).

### Modulation of chemotherapy-induced growth inhibition by indomethacin

To investigate growth inhibition after exposure to indomethacin and doxorubicin the MTT assay was used. GLC_4_-Adr cells are 190.6±16.2 times more resistant to doxorubicin as compared to GLC_4_. Two relatively nontoxic concentrations of indomethacin (10 and 20 *μ*M) that induced some caspase activation in GLC_4_-Adr, were used in combination with doxorubicin in an MTT assay. A dose of 10 and 20 *μ*M of indomethacin induced, respectively, 1 and 2% growth inhibition in GLC_4_, and respectively 15 and 17% in GLC_4_-Adr. In the presence of 20 *μ*M indomethacin there was no effect on doxorubicin sensitivity in GLC_4_, while a 2.7-fold increase in doxorubicin sensitivity was observed in GLC_4_-Adr (Table 1). Indomethacin, like doxorubicin, is also a substrate for the MRPl drug efflux pump, which is overexpressed in GLC_4_-Adr. We observed a similar increase in doxorubicin sensitivity comparing the effect of indomethacin with the effect of MK-571, a well-established inhibitor of MRPl, in the MTT assay (Table 1).

## DISCUSSION

This is the first study that illustrates the effective circumvention of doxorubicin resistance by indomethacin-induced activation of the death receptor apoptosis pathway in a doxorubicin-resistant SCLC cell line independent of Fas.

Fas-mediated apoptosis could only be induced in GLC_4_-Adr but not in GLC_4_ in the presence of the protein synthesis inhibitor cycloheximide, demonstrating that the death receptor-mediated apoptosis pathway is functional in the chemotherapy resistant cell line when a cellular block is removed. Interestingly, indomethacin induced apoptosis in GLC_4_-Adr but not in GLC_4_ in the absence of cycloheximide. No marked intensity differences were observed for pro- and antiapoptotic proteins involved in the mitochondrial apoptosis pathway. In contrast, several proapoptotic proteins important for the death receptor apoptosis pathway were higher expressed in the doxorubicin-resistant cell line GLC_4_-Adr compared to GLC_4_. For instance, the Fas-membrane expression was 3.1-fold higher in GLC_4_-Adr compared to the parental cell line. The higher Fas membrane expression may be due to the repetitive incubation with doxorubicin. It may also serve to facilitate a growth advantage to GLC_4_-Adr as was demonstrated in several Fas-positive tumour cell lines (Owen-Schaub *et al*, 1994; Siegel *et al*, 2000). The fact that the difference in Fas membrane expression does not correlate with the Fas expression in total cell lysates may be due to a different distribution of Fas, cytoplasmic and on the cell membrane, as was described in prostate carcinoma cell lines and neuroblastoma cell lines (Fulda *et al*, 1997; Usla *et al*, 1997; Bennett *et al*, 1998; Sodeman *et al*, 2000). The expression levels of the proapoptotic proteins caspase-8 and Bid were also elevated in GLC_4_-Adr compared to GLC_4_. Bid has been described to transport and recycle mitochondrial membrane phospholipids (Esposti *et al*, 2001). Since doxorubicin has toxic properties towards mitochondrial membranes, increased expression of Bid in GLC_4_-Adr may be an additional resistance mechanism. The sensitivity of GLC_4_-Adr to Fas-mediated apoptosis, in the presence of cycloheximide, as compared to GLC_4_ can therefore be due to the higher Fas-membrane levels as well as elevated expression levels of caspase-8 and Bid or a combination of these factors. Anti-Fas antibody alone induced minimal caspase-3 activation which was only slightly increased by combining it with doxorubicin in GLC_4_-Adr. Owing to the limited modulatory effects of doxorubicin on Fas-mediated apoptosis an alternative was sought.

The NSAID indomethacin has been identified as an apoptosis-inducing agent in different *in vivo* models and among the several mechanisms involved it can induce caspase-3-mediated apoptosis (Fujii *et al*, 2000; Kim *et al*, 2000; Sanchez-Alcazar *et al*, 2003). The apoptosis-inducing effect of indomethacin in GLC_4_-Adr is, however, not based on Fas/FasL interaction. Indomethacin did not affect Fas membrane expression and apoptosis is not decreased when cells are pretreated with an inhibiting anti-FasL antibody prior and during indomethacin exposure. Indomethacin alone induced extensive apoptosis in GLC_4_-Adr with activation of caspase-8, caspase-9 and PARP cleavage even at low doses. This did not occur in GLC_4_. The apoptosis-inducing effect of indomethacin will therefore most likely be due to a Fas receptor-independent effect on the death receptor-apoptosis pathway. However, we cannot exclude the involvement of other death receptors. Inhibition of either caspase-8 or caspase-9 by zIETD-fmk and zLEHD-fink, respectively, decreased indomethacin-induced apoptosis. Therefore, indomethacin-mediated apoptosis induction in the GLC_4_ cell lines depends on a functional mitochondrial apoptosis pathway, which is probably absent in GLC_4_ due to the decreased Bid expression. Indomethacin, however, did not decrease expression of Bcl-2, Bcl-X_S/L_ or Mcl-1 in GLC_4_ or GLC_4_-Adr which is in contrast to results described for lung adenocarcinoma cell lines (Lin *et al*, 2001). The feet that indomethacin can activate caspase-8, Bid and caspase-9 in GLC_4_-Adr makes it a good alternative for agonistic anti-Fas antibody. Indomethacin added to doxorubicin largely increased doxorubicin effects on caspase-3 activation and cytotoxicity in GLC_4_-Adr cells. Indomethacin, like doxorubicin, is a substrate for the MRP1 drug efflux pump, which is overexpressed in GLG_4_-Adr (Versantvoort *et al*, 1995; Draper *et al*, 1997; Touhey *et al*, 2002). Therefore, a subsequent increase in cellular doxorubicin concentration by indomethacin may have partly played a role. Other mechanisms by which indomethacin might induce apoptosis are increased glutathione extrusion mediated by MRP1 (Trompier *et al*, 2004) or increased ATP consumption by MRP1 ATPase activity in analogy to the observed verapamil-induced ATP consumption in P-glycoprotein-overexpressing cells (Broxterman *et al*, 1989).

The role of indomethacin in modulation of doxorubicin toxicity, however, cannot completely be explained by the MRP1 inhibitory effect. MK-571 is a far more effective inhibitor of MRP1-mediated drug efflux than indomethacin (Bagrij *et al*, 2001). Despite the similar fold of doxorubicin sensitisation with either drug, this suggests that the observed effect of indomethacin on doxorubicin sensitivity is due to an increase in drug accumulation as well as MRP1-dependent or independent caspase activation in GLQ-Adr cells.

Interestingly, the NSAIDs have recently caught much attention in the treatment of tumours in combination with chemotherapy to potentiate their effect (Gupta and DuBois, 2001). The first clinical report on a combination of celecoxib, an NSAID and selective cyclooxygenase-2 inhibitor, with chemotherapy appeared (Altorki *et al*, 2003). It showed an enhanced response to preoperative paclitaxel and carboplatin in early-stage non-small-cell lung cancer. This approach in SCLC may also be of interest not only because of Cox-2 inhibition but also because of the effect observed by us on the alternative apoptotic route compared to the route used by chemotherapy. The observed extensive potentiation of doxorubicin-induced inhibition of cell survival at achievable clinical doses indomethacin (Gupta and DuBois, 2001), deserves testing in the clinic. The potential effect of these concentrations of indomethacin on other chemotherapeutic drugs requires further testing in preclinical models.

Overall, it can be concluded that indomethacin increases the cytotoxic activity of doxorubicin in a doxonibicin-resistant SCLC cell line partly via the death receptor apoptosis pathway, independent of Fas.

## Figures and Tables

**Table 1:** A 50% inhibiting dose in the MTT assay (*μ*M) of doxorubicin and doxorubicin in combination with indomethacin (10 and 20 *μ*M) in GLC_4_ and GLC_4_-Adr

	**GLC_4_**	**GLC_4_-Adr**
Doxorubicin ( *μ*M)	0.014±0.001	2.750±0.050
Doxorubicin+indomethacin (10 *μ*M)	0.011±0.002	1.403±0.203^*^
Doxorubicin+indomethacin (20 *μ*M)	0.012±0.002	1.003±0.179^*^
Doxorubicin+MK571 (50 *μ*M)	0.014±0.002	0.743±0.121^*^

Data represent the mean±s.d. of three independent experiments (^*^P<0.01).
